# African Tick-bite Fever in French Travelers

**DOI:** 10.3201/eid1111.050852

**Published:** 2005-11

**Authors:** Paul H. Consigny, Jean-Marc Rolain, Daniel Mizzi, Didier Raoult

**Affiliations:** * Institut Pasteur de Paris, Paris, France; † Université de la Méditerranée, Marseille, France; ‡ Médecin de Santé au Travail, Plaisir, France; § Faculté de Médecine, Marseille, France

**Keywords:** rickettsioses, African tick-bite fever, travelers, letter

**To the Editor:** African tick-bite fever (ATBF) is caused by *Rickettsia africae* and remains the most common tickborne rickettsiosis in sub-Saharan Africa (*1**,**2*). We describe an outbreak of ATBF in 10 of 34 French tourists on their return from South Africa in March 2005. Fever, skin rash, and multiple eschars on the legs developed in the index case-patient (patient 9, Table). After informed consent was obtained, the tourists completed a questionnaire for epidemiologic and clinical data. Acute- and convalescent-phase serum samples were collected when possible for serologic analysis performed at the Unité des Rickettsies. The samples were tested against a panel of antigens including *R. typhi*, *Francisella tularensis*, *Coxiella burnetii*, *Borrelia burgdorferi*, *Anaplasma phagocytophylum*, *R. felis*, *R. helvetica*, *R. conorii* subsp. *conorii* strain Malish, *R. africae*, *R. sibirica mongolotimonae*, *R. massiliae*, and *R. slovaca*, as previously described (*3*). A case of symptomatic confirmed ATBF was defined as clinical illness and positive serologic results against *R. africae*, whereas a case of probable ATBF was defined as typical clinical symptoms without definite serologic evidence of R. africae infection.

**Table:** Epidemiologic, clinical, and serologic information for 10 patients with African tick-bite fever*

Patient	Sex/age (y)	Tick bite	Delay before onset (d)	Fever	Headache	Myalgia	Eschar (site)	Skin rash	1st serum† IgG/IgM	2nd serum† IgG/IgM	Diagnosis
1	M/62	No	7	Yes	No	No	Multiple (legs)	No	NA	NA	Probable
2	F/58	No	6	Yes	No	Yes	Multiple (legs, arms)	No	64/32	64/128	Confirmed
3	M/58	No	6	No	Yes	No	Single (trunk)	No	64/32	128/16	Confirmed
4	F/51	No	6	No	Yes	Yes	Multiple (legs, trunk)	No	0/64	128/16	Confirmed
5	M/58	No	5	Yes	No	Yes	Multiple (legs)	No	512/0	512/0	Confirmed
6	F/57	No	5	No	No	Yes	Yes (unknown)	Yes	NA	32/16	Confirmed
7	M/65	No	5	Yes	Yes	Yes	Multiple (hands)	No	128/64	512/128	Confirmed
8	F/59	No	10	No	No	No	Multiple (legs, arms, trunk)	No	64/8	128/32	Confirmed
9	M/53	No	3	Yes	Yes	Yes	Multiple (legs)	Yes	0/0	1,024/512	Confirmed
10	M/51	No	8	Yes	No	Yes	No	Yes	32/32	64/64	Confirmed
Total (%)		0		60	40	70	90	30			

*NA, not available; Ig, immunoglobulin; male-to-female ratio, 60%; mean age = 57.2 ± 4.5 years. †Identical results obtained with both *Rickettsia africae* and *R. conorii* antigens.

Of the 34 travelers, 30 completed the questionnaire and 20 consented to give at least 1 serum sample. After their return to France, symptoms compatible clinically with ATBF developed in 10 of the travelers (Table) and 9 had positive serologic results and/or a seroconversion for spotted fever group-rickettsia, including *R. africae* (Table). The median time from illness onset to serum testing was 19 days. Thus, 9 of the travelers had probable and 1 had possible (no serum was available) ATBF. Including both probable and possible cases, the illness rate for the whole group was 33.3% (10/30). None of the travelers reported a history of tick bite. The delay between probable exposure and onset of symptoms was 3-10 days (mean ± standard deviation 6.1 ± 1.9 days). Multiple eschars on the legs or arms were seen in 7 (70%) of 10 patients. Eight patients received doxycycline (200 mg per day) for a mean of 10.8 ± 5.9 days (range 5-20), 1 patient received pristinamycin for 8 days, and 1 patient received no treatment. All patients recovered fully without sequela; however, 6 patients reported convalescent-phase asthenia and 1 reported chronic insomnia, which had not occurred previously, for 2 months after the illness. Among the 10 remaining travelers, for whom a serum sample was available, with no clinical evidence of ATBF, 5 were positive for *R. africae* with only immunoglobulin M (IgM) at a titer of 1:32 in 4 cases and IgG at 1:128 with IgM at 1:32 in 1 case (an acute-phase serum from this patient showed IgG at 1:32 and IgM at 1:32). The 5 other travelers had negative serologic results. Results of serologic testing for other bacteria were negative for all travelers. Twenty-four travelers (80%), including the 10 symptomatic patients, reported using topical insect repellent daily.

Most cases of ATBF are reported in clusters of travelers exposed to ticks during game hunting or safaris, as described here (*1**,**3**-**5*). The estimated incidence of African tick-bite fever in safari travelers is 4%-5.3% (*4*) but higher incidence may be reported as emphasized in our study. In our study, epidemiologic and clinical data for the 10 symptomatic patients were obtained in accordance with current knowledge of ATBF (*2*).

Skin biopsy samples remain the best tool to isolate or detect *R. africae* (*2**,**6*). However, specific serologic tests, especially immunofluorescence assays, remain the most widely used microbiologic test worldwide (*7*). No commercially available test for ATBF exists but due to extensive cross-reactions between spotted fever group rickettsiosis, commercial kits based on the detection of R. conorii antibodies can be used for the diagnosis of ATBF. Most tourists reported using topical insect repellents without any efficacy. Applying repellents to exposed skin provides little protection against ticks because they can crawl underneath clothing and bite untreated portions of the body (*8*). Thus, treating clothing with synthetic pyrethroid insecticide is recommended to complement the topical repellant (*8*).

In conclusion, our study emphasizes the importance of ATBF as a common cause of flulike illness in travelers returning from South Africa, but with a higher rate than malaria, typhoid fever, or other tropical fevers. The most important clinical clues are the presence of clustered cases with multiple inoculation eschars. Healthcare professionals who are providing advice should inform persons traveling to endemic areas of Africa of the risk of contracting ATBF and the importance of protecting themselves against tick bites. Chemoprophylaxis with doxycycline is not recommended, however, this recommendation may be evaluated in future clinical trials.
